# Apoptosis stalks *Plasmodium falciparum* maintained in continuous culture condition

**DOI:** 10.1186/1475-2875-9-S3-S6

**Published:** 2010-12-13

**Authors:** Beth K Mutai, John N Waitumbi

**Affiliations:** 1 Walter Reed Project, Kenya Medical Research Institute, Kisumu, Kenya

## Abstract

**Background:**

Growth kinetic of *Plasmodium falciparum* in culture or in the host fall short of expected growth rate considering that there are 4 x 10^6^/µL red blood cell (RBCs) available for invasion and about 16 merozoites growing in each infected RBC. This study determined whether apoptotic machinery is operable to keep the parasite population under check.

**Methods:**

A synchronized culture of *P. falciparum* (Dd2 strain) was initiated at 0.5% ring stage parasitaemia and kept under conditions not limiting for RBCs and nutrient by adjusting hematocrit to 5% at each schizogony and changing growth media daily. Parasite growth pattern and morphology was evaluated by blood smear microscopy and flow-cytometry using SYBR green. The apoptotic processes were evaluated for evidence of: DNA fragmentation by TUNEL, collapse of mitochondria membrane potential (ΔΨ_m_) by TMRE, expression of metacaspse gene by RT-qPCR and by probing parasite proteins with anti-caspase antibodies.

**Results:**

From the seeding parasitaemia of 0.5%, the parasites doubled every 48 hours to a parasitaemia of 4%. Thereafter, the growth stagnated and the culture consistently crashed at about 6% parasitaemia. ΔΨ_m_ potential collapsed as the parasite density increased and DNA fragmentation increased steadily from 0.2% to ~6%. The expression of metacaspase gene and protein was observed in all stages, but their abundance was variable among the stages.

**Conclusion:**

These findings suggest existence of *P. falciparum* quorum sensing that keep the parasite population under check.

## Background

The development and propagation of malaria parasites in their vertebrate host is complex and involves various host and parasite factors. In absence of specific anti- parasitic immune responses such as in vitro culture conditions, it is reasonable to assume that the parasite growth rate reflects a balance between cell proliferation and cell loss. This assumption is strengthened by the following observations: 1) in vitro, the parasitaemia of *Plasmodium falciparum* cultures increases by a factor of 3-8 within 48 hours whereas theoretically it should increase 16 fold [1], and 2) in vivo, the evolution of parasitaemia in fast growing strains seems to be quelled by parasite density even when the RBC supply is not limiting [2]. It is tempting to speculate that the disparity between expected and observed parasite density is a function of self-regulation.

One of the physiological mechanisms of reducing cell number is by apoptosis [3], a process by which self inflicted morphological and biochemical changes result in cell death [4,5]. At the cellular level, apoptosis is characterized by controlled auto-digestion through the activation of endogenous proteases that result in cytoskeletal disruption, cell shrinkage, and membrane blebbing [6] Due to activation of endonucleases, the nucleus undergoes condensation as DNA is fragmented into oligonucleosomes. Formation of oligonucleosomes can be detected using terminal deoxynucleotidyl transferase-mediated desoxyuridine triphosphate (dUTP) nick-end labeling (TUNEL). [7] Even before DNA damage is evident, mitochondria show collapse in the electrochemical gradient as measured by change in the mitochondrial membrane potential (ΔΨ_m_). Changes in ΔΨ_m_ lead to the insertion of proapoptotic proteins into the membrane resulting in pore formation and subsequent release of cytochrome c into the cytoplasm. [6] Loss of ΔΨ_m_ can be measured by a cell-permeant, cationic, red-orange fluorescent dye Tetra methyl Rhodamine ethyl ester (TMRE) that is readily sequestered by active mitochondria [8] or cationic dye 5,5,6,6’-tetrachloro-1, 1,3,3’-tetraethylbenzainidazolocarbocyanin iodide, commonly known as JC-1 [9].

For a long time, apoptosis was assumed to be confined to metazoan organism but recent findings indicate that unicellular organisms also undergo apoptosis and features typical of mammalian programmed cell death have been described in several species including the kinetoplastids: *Trypanosoma brucei rhodesiense, Trypanosoma cruzi* and *Leishmania major*[7,10-15]. Features typical of apoptosis including condensation of chromatin, DNA fragmentation, the appearance of phosphatidyl serine (PS) on the outer leaflet of the plasma membrane and eventual formation of apoptotic bodies have been demonstrated in *Plasmodium berghei*[16]. Picot *et al*[17] previously reported oligonucleosomal DNA fragmentation in the erythrocytic stages of chloroquine treated *Plasmodium.* Recently, DNA fragmentation and mitochondrial membrane potential disruption have been shown in erythrocytic stages of 3D7 and 7G8 isolates of *P. falciparum* treated with apoptosis- inducer etoposide or anti-malarial chloroquine [17,18]. Although clear homologues of metazoan caspases have not been identified in *Plasmodium*, two cysteine proteases have been annotated (PF13-0289 and PF- 0363) in the *P. falciparum* genome database [19]. The two proteases have 24% sequence identity and have the conserved catalytic dyad histidine and cysteine required for catalysis activity and contains a putative caspase recruitment domain in the N-terminal amino acid sequence [18]. Presence of caspase-like proteins in *Plasmodium* was also suggested by use of caspase inhibitors (Z-VAD.fmk (Benzyloxycarbonyl-Val-Ala-Asp flouromethylketone) and Z.DEVD.FMK (Benzyloxycarbonyl-Asp-Glu-Val-Asp- flouromethylketone)) that inhibited *Plasmodium* apoptosis leading to suggestion that caspase-like activity and aspartate specificity may be crucial for apoptosis in *Plasmodium*[16]. In addition to metacaspase gene, an apoptosis related gene (PfARP) has been purified and characterized in *P. falciparum* although the role of this protein in growth, multiplication and stage progression has not been identified [20].

Existence of apoptosis in protozoa suggests that even these unicellular organisms live as communities where communication occurs between individual cells. This interaction between protozoa is similar to the quorum sensing (QS) and biofilm formation in bacteria [21,22]. QS as is known in bacteria, refers to regulation of gene expression in response to fluctuations in cell population density and is mediated by signaling molecules released into the environment. In vitro studies and epidemiological data provide evidence that malaria parasites exhibit QS [23]. The process relies on production of low molecular mass-signaling molecules called auto-inducers, the extra-cellullar concentration of which is related to the population density [23].

Although *P. falciparum* could regulate parasite density by varying the rate of cell division or varying the rate of invasion of RBC, its been suggested that varying the rate of cell death is the most effective of these mechanisms because it slows down the increase of the parasitaemia while at the same time decreasing the percentage of infected RBCs independent of the host’s immune system [1].

In this study, density dependent processes that limit expansion of the malaria parasites are described. It is possible that these processes may one day be exploitable to provide new strategies of curtailing disease severity associated with parasite density.

## Methods

### Study design

A Dd2 clone of *P. falciparum* was obtained from cryopreserved stocks maintained at the KEMRI/Walter Reed Project - Kenya, and used to initiate culture growth. Highly synchronized cultures (>98% young rings) were allowed to grow in conditions that were not limiting in RBCs and nutrients. At every developmental stage, aliquots were taken and used to assess parasitaemia, parasite morphology and presence of selected apoptotic features. A sham culture of uninfected RBCs was maintained in a similar manner to the parasite culture and was used as background noise for the target apoptotic features.

### Maintenance of *P. falciparum* cultures

*Plasmodium falciparum* cultures were maintained using continuous culture conditions [24] with minor modifications. A clone of *P. falciparum* Dd_2_ isolate was used to initiate parasite growth in washed group O^+^ human erythrocytes diluted to 5 % hematocrit in complete RPMI 1640 media supplemented with 0.2% bicarbonate, 25 mM HEPES, 50 µg/ml gentamicin and 10 % heat inactivated human serum. Cultures were maintained in 25 cm^2^ Corning flasks (Corning incorporated, Corning NY, USA), with daily replacement of growth medium to meet the nutrient requirements and maintain optimal pH.

Synchronized cultures were obtained by enriching for young ring stage trophozoites using 5% D- sorbital which lyses RBCs containing late rings stage and other mature parasites stages [25]. This treatment was repeated every 48 hours until >98% of the parasites were synchronized in the ring stage as confirmed by microscopy.

### Parasite growth kinetics by light microscopy and flow cytometry

Following initiation of the culture at parasite density of 0.5%, at every developmental stage and subsequent parasitaemia levels, 10 µL aliquots was removed and used for Giemsa-stained thin blood film microscopy for monitoring: 1) the level of parasitaemia by counting a minimum of 10,000 erythrocytes and 2) the morphological changes in the parasites.

Percentage parasitaemia was also scored using SYBR Green (Molecular Probe, Eugene, OR, USA), a cell permeable dye that stains nucleic acids. 100 µl of parasite culture at 2% haematocrit suspension was incubated with 200 µl of 1x SYBR Green (supplied as 10,000x) for 30 minutes at room temperature. The cells were washed three times in PBS and then resuspended in 400 µl PBS in FACs tube. Uninfected RBCs from the sham culture were stained in a similar manner as the parasites and used to set acquisition gate. 20,000 events were acquired on FL1 and subsequently analyzed on a FACScan (Becton Dickinson, San Jose, CA, USA) using the FlowJo software.

### DNA fragmentation assay

TUNEL method (in situ cell death Detection kit, Roche Diagnostics, Germany) was used to probe for DNA fragmentation. 500 µl of the cell suspension at a concentration of 20,000 cells/µL was concentrated on a slide by centrifuging in a cytospin (Shandon cytospin 4, Thermo Electron Corporation, USA) at 500 rpm for 3 minutes. The cells were fixed with 4 % Para formaldehyde and then stained according to the kit manufacturer’s protocol. For each sample, a positive and a negative control sample was included. For a positive control, the sample slide was incubated with DNase 1 (Invitrogen, Carlsbad, CA) at a final concentration of 0.003 U /µL in DNase buffer (50 Mm Tris-HCL, pH 7.5,1 mg /ml BSA) for 10 minutes at 25 °C. The slides were rinsed in PBS to remove excess DNase 1. For the negative control the slide was incubated without terminal transferase enzyme. Slides were observed under a BX40 fluorescence microscope (Olympus Melville, NY) equipped with a Magnafire digital camera. For analysis by flow cytometry, 100 µL of paraformaldehyde fixed cell suspension at 20,000 cells/ µL was stained as above. Control samples were as described above. 20,000 events were acquired on FL3 (FACScan, Becton Dickinson, San Jose, CA) and analyzed using the cellquest Pro. Version 5.2.

### Monitoring of mitochondrial membrane potential

Collapse of ΔΨ_m_ is a hallmark of apoptosis and mitochondrial permeability is an important step in induction of cellular apoptosis. The ΔΨ_m_ collapse was monitored using TMRE (Invitrogen, Eugene Oregon, USA), a cell permeable cationic dye that is accumulated by health cells in the mitochondria in proportional to mitochondrial potential.

For this assay, an aliquot of 100 µL of cell culture was incubated with 25 nM TMRE for 15 minutes at 37 ° C in the dark. The cells were then washed twice in PBS and resuspended to a final volume of 400 µL and analysed on FL2 by flow cytometry. Uninfected RBCs from the sham culture were stained in a similar manner as the parasites and used to set acquisition gate. 20,000 events were acquired on FL2 and subsequently analysed on a FACScan (Becton Dickinson, San Jose, CA, USA) using the FlowJo software.

### Analysis of metacaspase gene expression

RNA was isolated using commercially available kit as recommended (Invitrogen, life technologies, California). To ensure that equal number of iRBCs weres used for RNA isolation at the different parasite densities, 100 µL of well suspended culture cells was run on a Coulter Counter (Ac. T 5diff CP, Beckman coulter Inc. Miami Florida. USA) to obtain RBCs count per µL. Numbers of iRBC were calculated from parasitaemia data obtained from microscopy and for each sample, 5 x 10^6^ IRBCs were used.

A one–step RT-PCR (Applied Biosystem, Roche, Branchburg, New Jersey. USA) was used for simultaneous analysis of *P. falciparum* metacaspase and seryl tRNA synthetase gene transcript. The latter is a *P. falciparum* house keeping gene that is expressed equally in each stage of the *P. falciparum*[26] and was used for normalization of metacaspase gene transcripts. Nucleotide sequences for metacaspase and seryl tRNA were obtained from PlasmoDB library and used to design primers using Primer Express 3.0 software (Applied Biosystems, California. USA). The following primer pairs were from Applied Biosystems were use: Metacaspase primers: forward 5’-CAT CCT TGT CCC ATC AAT CTT TT-3’, reverse 5’-ATG GAA ACC CTC CTT AAA ATT AG-3’. Seryl tRNA synthetase primers: forward 5’-TAT CAT CTC AAC AGG TAT CTA CAT CTC CTA-3’, reverse 5’-TTT GAG AGT TAC ATG TGG TAT CAT CTT TT-3’. Reverse transcription and amplifications were done on a ABI PRISM 7300 PCR system (Applied Biosystems, California. USA). The PCR reactions were carried out in 96-well plate (Applied Biosystems Cat. No.4306737) each containing 25 µL Power SYBR Green PCR master mix (1.5 mM MgCl_2,_ 0.2 mM of each dNTPs, 1.25 U/µL AmphliTaq Gold DNA polymerase), 0.25 U/µL multiscribe reverse transcriptase, 0.4 U U/µL RNAse inhibitor, 2.5 µL RNA, 0.9 µM metacaspase primers, 1 µM seryl tRNA synthetase primers and PCR water to a final volume of 50 µl. The PCR mix also contained ROX dye as an internal reference. Reverse transcription step was done at 48 °C for 30 minutes followed by heat inactivation of reverse trancriptase enzyme at 95 °C for 10 minutes. The amplification was then done for 45 cycles of 95 °C for 15 seconds and 60 °C for 1 minute. A dissociation analysis step was included to check the specificity of the amplification.

### Analysis of metacaspase protein by Western blotting

To ensure that equal number of iRBCs was used for protein extraction at different parasite densities, 100 µL of well-suspended culture cells was run on a Coulter Counter and numbers of iRBCs calculated as described earlier. After working out the volume of the culture required to give the required number of iRBC, the specified volume was removed and pelleted by centrifugation at 3,000 rpm for 3 minutes at 4 °C. The pellet was resuspended to 100 µL using culture media devoid of serum and stored at -80 °C until all the samples were ready for analysis.

Equal volume of 2X SDS sample buffer (100 mM Tris-HCL, pH 6.8, 4 % SDS, 0.2 % bromophenol and 25 % gycerol) was added the lysed cells and then resolved by electrophoresis on an 12 % SDS-PAGE gel (5% stacking) at 120 volts for 1 hour. The protein was then electro-blotted onto the nitrocellulose membrane (Invitrogen, Carlsbad, CA). Thereafter, the membrane was blocked at 4 °C overnight with gentle agitation using 1X casein in PBS containing 10 % Tween-20. Thereafter, the membrane was incubated for 1 hour with 1:500 dilution of rabbit polyclonal anti-human caspase-3 or 7 (Enzo Life Sciences International, NY, USA). The membranes were then washed three times in PBS containing 10 % Tween-20 and then incubated for 1 hour at room temperature with 1:20,000 dilutions of anti-rabbit IgG conjugated to horseradish peroxidase in 1X casein. The blots were washed as indicated above and developed by chromogenic peroxidase reaction with 3, 3’ – diamino benzidine (Pierce Biotechnology, Inc., Rockford, IL, USA) before being exposing to a Kodak Biomax film (Rochester, NY, USA) for 30 seconds.

## Results

### Growth characteristics of *P. falciparum* Dd2 isolate

**Figure 1:**
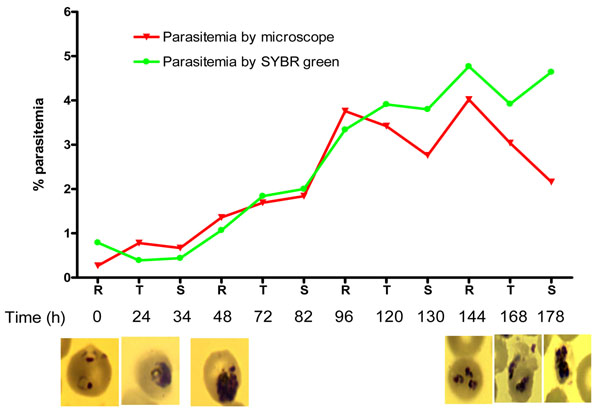
**In vitro growth pattern of *P. falciparum* Dd2 isolate as measured by microscopy (red curve and photomicrograph) and SYBR Green (green curve).** By both methods, parasitaemia initiated at 0.5% doubled at every schizogony and did not increase beyond 5%. **The photomicrograph show** healthy parasite at seeding parasitaemia: note the classical signet ring, healthy looking trophozoites with irregular borders and mature schizonts with numerous nuclei. At maximal parasitaemia, the ring stages were smaller with intensely stained nuclei while the trophozoites and schizonts have abnormal nuclei.

The growth curve of a highly synchronized Dd2 isolate is shown in Figure 1. Parasites seeded at a starting ring stage parasitaemia of ~ 0.5% doubled after every schizogonic cycle (48 hours) to a maximum parasitaemia of 5%. Thereafter, the growth stagnated as determined by microscopy and SYBR Green measurements by flow-cytometry. However, while parasitaemia count by microscopy declined steadily after day 6 of culture, parasitaemia by SYBR Green remained slightly elevated.

### DNA fragmentation increases with rise in parasitaemia

**Figure 2:**
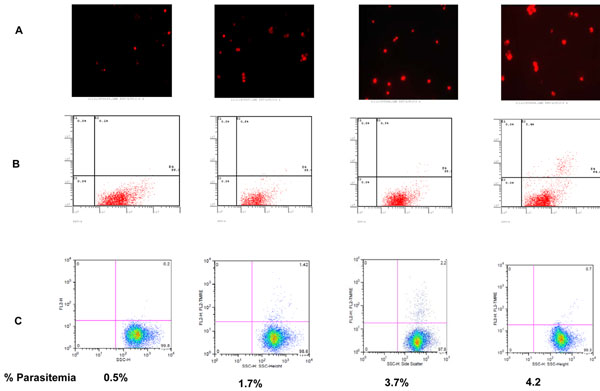
**Panel A and B: Level of fragmented DNA using terminal deoxynucleotidyl-transferase-mediated dUTP nick-end labeling (TUNEL).** Evidence of DNA fragmentation as measured by TUNEL is discernible albeit at very low level by fluorescent microscopy (Panel A) and flow cytometry (Panel B) at seeding parasitaemia, but increases dramatically by the time the parasite culture crashes. **Panel C: Dot blots showing percentage accumulation of TMRE in the mitochondria of trophozoites at different parasitaemia.** As shown, the proportion of cells with positive TMRE staining was maximal at 3.7% parasitaemia and declined thereafter.

DNA fragmentation, a characteristic feature of cells undergoing apoptosis can be revealed by assessing incorporation of end-labeled nucleotides to the exposed 3’-OH terminal of DNA ends. As shown in Figure 2, DNA fragmentation was evident by both fluorescent microscopy (photomicrograph, panel A) and flow cytometry (dot blots, panel B) at the seeding parasitaemia (0.5%) and thereafter. As would be expected from the number and size of nuclei, trophozoite and or schizont stages showed more pronounced DNA fragmentation than the ring stages. DNA degradation was especially high for schizonts at maximum parasitaemia.

### Loss of mitochondrial membrane potential increases with rise in parasitaemia

Changes in the mitochondrial ΔΨ_m_ is a key feature of apoptosis and can be revealed by staining with TMRE, a cell permeable cationic dye that accumulates in the mitochondria of health cells. During apoptosis, mitochondrial become permeable leading to loss of ΔΨ_m_ and accumulation of TMRE is reduced. Figure 2C shows accumulation of TMRE as parasite density increases. As shown, the proportion of cells with TMRE was maximal at 3.7% parasitaemia and declined thereafter indicating increased permeability of mitochondria membrane and subsequent loss of TMRE.

### Expression levels of metacaspse gene

**Figure 3:**
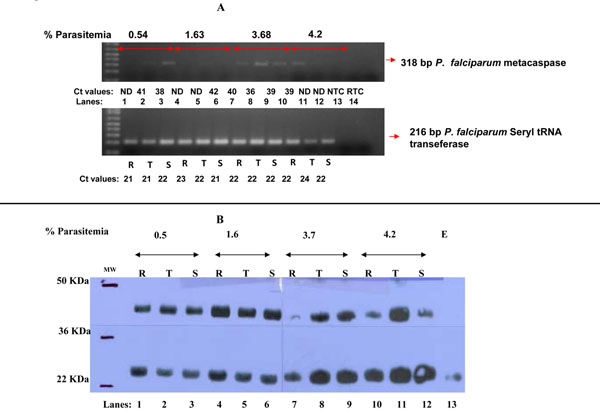
**Panal A: Agarose gel picture showing expression level of metacaspase and Seryl transferase genes in rings (R), trophozoites (T) and schizonts (S) at different parasite densities (double arrow bar).** NTC= No template control (Lane 13 ). RTC= Reverse transcription control (lane 14). Expression of metacaspase gene was stage dependent. At the ring stage metacaspase was absent until the parasite density reached 4%. For the trophozoites, the metacaspase gene was detectable at 0.54% and 3.68% parasitaemias while in the schizonts, the gene was present at all parasitaemia levels. The *P. falciparum* house keeping gene, seryl tRNA, was expressed at relatively equal level in all the RNA preparations. **Panel B: Western blot analysis of *P. falciparum* protein extract probed with anti- caspase-7 polyclonal antibody.** Protein fragments of 45 and 28 kDa were observed in all the developmental stages (lanes 1-12). Metacaspase protein was up-regulated starting from parasite density of 1.6% in all the parasite stages and remained up-regulated in subsequent parasitaemia levels except in the ring (lanes 7 and 10). Note a low levels of protein of 28 kDa in uninfected erythrocytes (lane 13) and has been reported before in other negative control samples. [16]

To assess how increase in parasite density affects expression of metacaspase gene, a house keeping gene, seryl tRNA was used as the normalizing control for the target gene. As the Ct values of the seryl tRNA gene indicate, there was an equal amount of mRNA in all the preparations (Figure 3, panel A). Metacaspase gene expression was stage dependent and in general (see Ct values in Figure 3, panel A), and barely detectable on agarose gel (Figure 3, panel A). For ring stage, metacaspase was absent until the parasite density reached 4.2%. For the trophozoites, the metacaspase gene was detectable at seeding parasitaemia and at 3.7%, while in the schizonts the gene was expressed at all parasitaemia levels.

### Translation profiles of P. falciparum metacaspase protein

Figure 3, panel B shows a western blot of malaria parasite protein probed with anti-caspase 7 polyclonal antibody. The expected fragments of 45 and 28 kDa were observed in all the developmental stages (lanes 1-12), a pattern absent from uninfected erythrocyte preparation. A protein of approximately 28 kDa was present in uninfected erythrocytes (lane 13) and has been reported before in other negative controls [2].

Judging from protein band intensity, metacaspase protein was up-regulated at parasite density of 1.6% in all the developmental stages and remained up-regulated in subsequent parasitaemia except in the ring stages (lanes 7 and 12).

## Discussion

Like in multicellular organisms where apoptosis is essential for maintaining order and harmony among cells that must live together, protozoan parasites growing and differentiating in culture, in insect vector or in mammalian host display a structured organization that suggest that they ‘talk’ to each other and influence how they interact with elements of their environment [27]. Some of the factors used for “talking” have been identified as derivatives of homoserine lactone (HSL), which on secretion and at a threshold concentration induces a common genetic response in the population. [28] Such factors have been identified in protozoan organisms such as *Dictyostelium*[29]*, Plasmodium*[30] and *Trypanosoma brucei*[12].

Studies of free-living bacteria and single-celled organisms have shown they are capable of a form of quorum sensing whereby all individual cells secrete a signal factor. Such factors are derivatives of homoserine lactone which have been shown to induce a common genetic response in the population once a threshold concentration is reached [28].

In this study, in order to determine whether apoptosis triggered the events that were observed in the growth curve of the Dd2 parasite populations used in this study, a set of experiments were conducted to examine the use of apoptotic machinery at various stages of the parasite growth. In this experimental set up, parasitaemia increased by a factor of 1.5 to 2 every 48 hours, while the theoretical growth has been estimated to be by 16-32 folds [26]. Thereafter, the culture crashed when the nutrients or red blood cells were not limiting (Figure 1). Early in the growth curve, the morphological features reviewed by microscopy were those of healthy malaria parasites [32,33]. Parasites with compromised morphological features were seen later as parasitaemia increased (Figure 1).

An important molecular indicator of apoptosis is DNA fragmentation whereby the DNA is degraded at the internucleosomal regions by endogenous DNAses into fragments of 180-200 base pairs [34]. The degraded DNA can be revealed by electrophoretic separation of these fragments in agarose gel or by incorporating labeled nucleotides to the exposed 3’-OH terminal of nicked DNA ends. Using the latter method, evidence of DNA fragmentation was discernible albeit at very low level by flow cytometry and by fluorescent microscopy at the seeding parasitaemia and increased dramatically by the time of parasite culture crash (Figure 2, panel A and B). Most of the DNA degradation was evident at the trophozoites and schizont stages, a finding that probably reflects increased cellular DNA content in these two stages [35]. These findings represent the first report of density dependent DNA fragmentation in asexual stages of *P. falciparum*. Previously reports showed DNA fragmentation following chloroquine treatement [17] and in the ookinates of *P. berghei.*[16].

During apoptosis, death stimuli induce mitochondrial membrane permeabilization that results in loss of mitochondria membrane potential. This loss can be revealed as decline of TMRE staining of the mitochodria. As shown in Figure 2, panel C, the proportion of cells with intact mitochondria declined as parasite density increased and was maximal at a day before the crash. The uptake of TMRE in the mitochondria varied among the stages, with the ring showing the greatest mitochondrial response (Figure 2, panel C). This difference in uptake of TMRE is probably as a result of differing level of metabolic activities in the three asexual blood stages [36], although further biochemical evidence will be required to validate this observation. From MedLine literature search, this is the first report in on the use of TMRE to study depolarization of mitochondria in *P. falciparum*.

The next evaluation examined the expression level of *P. falciparum* metacaspase gene and/or protein in relation to changes in parasite density. The metacaspase gene was expressed in all the asexual stages (Figure 3, panel A). Large variations in levels of gene expression in the different parasite stages were observed at different parasite densities (Fig 3, Panel A). It was confirmed that the seeming differential expression of this gene was not due to variation in quantity or quality of RNA by parallel analyses of expressing level of a house keeping gene, the seryl tRNA gene. A reverse transcription control was also used to confirm that amplification was not from genomic DNA. For ring stages, metacaspase was not expressed until the parasite density of 4% was attained (Figure 3, panel A, lane 8). While the expression of metacaspase gene was only evident in the trophozoites at 0.5% and 4% parasitaemia, in the schizonts, metacaspase was present at all parasitaemia levels (Figure 3, panel A). We are not sure what the cause of this large variation in the transcripts is, but could reflect changes in mRNA stability as the parasites proceed through different development stages.

Since gene transcriptional activity does not necessarily translate into protein expression, [37]changes in metacaspase protein expression as parasite density changed were also determined. The proteolytic activation of caspases is highly conserved in higher eukaryotes and depends on prodomain removal and cleavage of protein fragments [38]. Using anti- caspase 3 and 7 polyclonal antibodies to probe for *P. falciparum* metacaspase, two protein fragments of 45 kDa and 28 kDa were detected in all the asexual stages (Figure 3, B) as has been described before [16]. The expression level of metacaspase protein increased with rise in parasite density (Figure 3, B, lanes 8-12). A low level of protein of approximately 28 kDa was present in uninfected erythrocytes (lane 13) and has been reported before in other negative control samples [16]. Therefore this protein is from un-infected RBCs.

The four key indicators of apoptosis, namely DNA fragmentation, collapse of mitochondrial potential, up-regulation of metacaspase gene and protein transcripts accompanied collapse of malaria in vitro culture that was maintained under conditions that were not limiting for nutrients or red blood cells. These findings and others fro earlier studies are indicative of presence of a density dependent process for limiting uncontrolled growth of malaria parasites. These findings offer important insights that may in future be exploitable as chemotherapeutic targets.

## Competing interests

The authors declare that they have no competing interests.

## Authors' contributions

BKM conducted all the experiments, helped in data analysis and edited the manuscript. JNW designed the study, directed the work and drafted the manuscript. All authors have read and approved the final manuscript.
